# Spicy food intake predicts Alzheimer-related cognitive decline in older adults with low physical activity

**DOI:** 10.1038/s41598-023-35234-0

**Published:** 2023-05-16

**Authors:** Jaeuk Hwang, Young Min Choe, Guk-Hee Suh, Boung Chul Lee, Ihn-Geun Choi, Jun Ho Lee, Hyun Soo Kim, Shin Gyeom Kim, Dahyun Yi, Jee Wook Kim

**Affiliations:** 1 Department of Psychiatry, Soonchunhyang University Hospital Seoul, Seoul, 04401 Republic of Korea; 2 Department of Neuropsychiatry, Hallym University Dongtan Sacred Heart Hospital, 7 Keunjaebong-gil, Hwaseong-si, Gyeonggi-do 18450 Republic of Korea; 3 Department of Psychiatry, Hallym University College of Medicine, Chuncheon, Gangwon 24252 Republic of Korea; 4 Department of Neuropsychiatry, Hallym University Hangang Sacred Heart Hospital, Seoul, 07247 Republic of Korea; 5 Department of Psychiatry, Seoul W Psychiatric Office, Seoul, 07247 Republic of Korea; 6 Department of Neuropsychiatry, Seoul National University Hospital, Seoul, 03080 Republic of Korea; 7 Department of Laboratory Medicine, Hallym University Dongtan Sacred Heart Hospital, 7 Keunjaebong-gil, Hwaseong-si, Gyeonggi-do 18450 Republic of Korea; 8 Department of Neuropsychiatry, Soonchunhyang University Bucheon Hospital, Bucheon, 14584 Republic of Korea; 9 Institute of Human Behavioral Medicine, Medical Research Center, Seoul National University, Seoul, 03080 Republic of Korea

**Keywords:** Biomarkers, Epidemiology, Translational research

## Abstract

A plausible association exists among spicy food consumption, physical activity, and Alzheimer’s disease (AD) or cognitive decline, but it remains poorly investigated. We aimed to examined the association between spicy food and AD-related memory decline or global cognitive decline in older adults under the moderating effect of physical activity. Total 196 non-demented older adults were included. Participants underwent comprehensive dietary and clinical assessments including spicy food intake, AD-related memory, global cognition, and physical activity. The strength of spicy food was stratified into three categories: ‘not spicy’ (reference), ‘low spiciness’, and ‘high spiciness’. Multiple linear regression analyses were performed to examine the relationships between spicy level and cognition. The spicy level was the independent variable in each analysis; it was entered as a stratified categorical variable using the three categories. We found a significant association between a high level of spiciness in food and decreased memory ($\beta$ − 0.167, *p* < 0.001) or global cognition ($\beta$ − 0.122, *p* = 0.027), but not non-memory cognition. To explore the moderating effects of age, sex, apolipoprotein E ε4 allele-positivity, vascular risk score, body mass index, and physical activity on the associations between spicy level and memory or global cognition, the same regression analyses were repeated including two-way interaction terms between the spicy level and each of the six variables as an additional independent variable. An interactive effect was detected between a high level of spiciness in food and physical activity on the memory ($\beta$ 0.209, *p* = 0.029) or global cognition ($\beta$ 0.336, *p* = 0.001). Subgroup analyses showed that the association between a high level of spiciness in food and a lower memory ($\beta$ − 0.254, *p* < 0.001) and global score ($\beta$ − 0.222, *p* = 0.002) was present only in older adults with low physical activity, but not in older adults with high physical activity. Our findings suggest that spicy food intake is predictive of AD-related cognitive decline, i.e., episodic memory; this relationship is worsened by physically inactive lifestyle.

## Introduction

A spicy taste is a sensation of heat caused by the activation of pain receptors in the human tongue^1,2^. Some foods with unique tastes (e.g., sweet and salty foods) seems to be associated with Alzheimer’s disease (AD) and cognitive decline^3–5^; however, any association between the consumption of spicy food and AD or related cognitive dysfunction remains uncertain. Some previous preclinical and human studies found that spicy food improves AD and related cognitive dysfunction^6–8^. In contrast, a 15-year longitudinal, population-based cohort study found that consuming more spicy food was positively associated with lower cognitive scores, as measured by the global cognitive score and self-reported memory loss^9^. Additionally, preclinical studies have demonstrated that high capsaicin intake is associated with a neuronal, glutamate excitotoxic effect and an analgesic effect mediated via denervation of sensory nerves^10–13^.

Many studies have shown that physical activity has neuroprotective effects on the brain via various mechanisms^14^. A recent preclinical study of the mouse cerebral cortex revealed that physical activity lowers excessive brain glutamate levels in the brain and reduces its toxicity by increasing mitochondrial glutamate oxidation^15^. A meta-analysis found that physical activity was associated with a decreased incidence of AD^16^. Two prospective cohort studies found that physical activity was associated with better cognitive function, less cognitive decline, and a lower incidence of dementia^17,18^.

Despite possible associations between consumption of spicy foods, physical activity and Alzheimer's disease or cognitive decline, previous studies have investigated only the association between spicy food and physical activity^6–9,16–18^. No study has yet investigated other possible relationships or the moderating effects of their interactions. A human study showed that the effect of a Mediterranean-type diet on AD burden may vary by physical activity: participants not on such a diet with low physical activity had the highest AD burden, and those on such a diet who engaged in high physical activity had the lowest AD burden^19^. Such findings suggest interactions among spicy foods, physical activity, and AD-related cognitive decline.

Therefore, we examined the association between spicy food intake and AD-related memory decline or global cognitive decline in older adults without dementia. We explored the moderating effect of physical activity on the associations and determined whether physical activity affected on the associations between spicy food intake and AD-related memory decline or global cognitive decline.

## Methods

### Study design, setting and sample

The present study enrolled 196 adults without dementia (age between 65 and 90 years): 113 cognitively normal (CN) and 83 mild cognitive impairment (MCI) adults. The participants were recruited among individuals who attended in a dementia screening program at the memory clinic of Hallym University Dongtan Sacred Heart Hospital, Hwaseong, Republic of Korea^2^. Volunteers were invited for an eligibility assessment and additional volunteers from the community were recruited through recommendations from other participants, family members, friends, or acquaintances^2^. The CN group consisted of participants with a Clinical Dementia Rating^20^ score of 0 and no diagnosis of MCI or dementia^2^. All participants with MCI met the current consensus criteria for amnestic MCI, including memory complaints confirmed by an informant, objective memory impairment, preservation of global cognitive function, independence in functional activities, and the absence of dementia^2^. The age-, education-, and sex-adjusted z-score was < − 1.0 for at least one of the four episodic memory tests included in the Korean version of the Consortium to Establish a Registry for Alzheimer’s Disease (CERAD) neuropsychological battery, such as word list memory, word list recall, word list recognition, and constructional recall tests^2,21–23^. All participants with MCI had a Clinical Dementia Rating score of 0.5^2^. The exclusion criteria were the presence of a major psychiatric disorders, a significant neurological or medical illness, or a comorbidity that could affect cognitive functioning; illiteracy; visual/hearing difficulties or severe communication or behavioral problems that could make clinical examinations difficult; and the use of an investigational drug^2^.

### Assessment of spicy level and other dietary habits

Participants were systematically interviewed for spicy foods they had eaten. To assess the strength of spicy food consumed among participants who ate spicy food at least once per week during the past year, participants were asked to indicate the age at which they began eating spicy food, as well as the strength of spicy food that they usually preferred.

The strength of spicy foods consumed were assessed by trained researchers in accordance with previously described methods^24^, using the following international standard scales for spicy levels in all forms of spicy foods: i.e., Scoville heat units (SHU), part-per-million (ppm) of capsaicin, and Gochujang hot taste units (GHU) for the hotness of Korean red chili paste or gochujang (https://en.wikipedia.org/wiki/Scoville_scale; https://en.wikipedia.org/wiki/Gochujang; https://e-ks.kr/streamdocs/view/sd;streamdocsId=72059204116582329). As most Korean spicy foods are marked in units of spiciness i.e., SHU, ppm, and GHU, the intensity of spiciness was obtained through in-depth interviews. The strength of spicy food was stratified into three categories: ‘not spicy’, ‘low spiciness’ (300–900 SHU; 20–60 ppm; levels 1 and 2 [mild to slightly hot] in GHU), and ‘high spiciness’ (≥ 900 SHU; ≥ 60 ppm; levels 3, 4, and 5 [medium to extremely hot] in GHU). A no spiciness level was used as a reference; the spicy levels were categorized into two groups based on the median quartile (low spiciness group vs. high spiciness group), consistent with the categorization based on the mean capsaicinoid content of foods commonly consumed in the Republic of Korea^25^. All participants were systematically interviewed to identify other dietary patterns (i.e., consumption of proteins, vegetables, fruits, salty foods, fatty foods, and fried foods) selected from the mini-dietary assessment^26^. The mini-dietary assessment tool was devised based on dietary guidelines and the food pyramid for Koreans; it provides a valid evaluation of overall dietary habits, including food components and dietary regulation of the diet^27^.

### Clinical assessments

All participants underwent standardized clinical assessments by psychiatrists with more than 10 years of clinical experience based on the clinical assessment protocol, which incorporates the CERAD clinical and neuropsychological battery^21,22^. Trained neuropsychologists administered the neuropsychological assessment protocol incorporating the CERAD neuropsychological battery^23^ to all participants. AD-related cognitive domain was measured by the episodic memory, as the earliest cognitive change in AD^28–33^, and non-memory cognition for comparative purposes. The episodic memory score was determined by summing the scores of the four episodic memory tests (word list memory, word list recall, word list recognition, and constructional recall) in the CERAD neuropsychological battery. The non-memory score was calculated by summing the scores of the three non-memory tests (verbal fluency, modified Boston naming test, and constructional praxis) in the CERAD neuropsychological battery. The TS was generated by summing the scores of the seven tests in the CERAD neuropsychological battery, including verbal fluency, modified Boston naming, word list memory, constructional praxis, word list recall, word list recognition, and constructional recall^34^. Physical activities were evaluated using the Korean-version of the Physical Activity Scale for the Elderly (PASE) ^35,36^, which has been tested for reliability and validity. Trained neuropsychologists assessed the frequency, duration and intensity of activities that participants performed with respect to leisure, household, and occupational activities during the previous week. The test items were weighted and summed to obtain a total physical activity score based on PASE sub-scores reflecting leisure, household, and occupational activities. A higher score indicated greater physical activity. Participants were categorized into two the physical activity groups based on the median quartile (high physical activity group vs. low physical activity group). Vascular risks (e.g., hypertension, diabetes mellitus, dyslipidemia, coronary heart disease, transient ischemic attack, and stroke) were assessed based on data collected by trained researchers during systematic interviews of participants and their family members^2^. The vascular risk score (VRS) was computed based on the number of vascular risk factors present and was reported as a percentage^2,37^. The Geriatric Depression Scale was used to measure the severity of depressive symptoms^2,38,39^. Annual income was categorized into below the minimum cost of living (MCL), above the MCL but below twice the MCL, and at or above twice the MCL (http://www.law.go.kr)^2^. The MCL was determined from data published by the Ministry of Health and Welfare of the Republic of Korea in November 2012; it was 572,168 Korean Won (equivalent to 507.9 United States dollars) per month for a single-person household with an additional 286,840 Korean Won (equivalent to 254.6 United States dollars) per month for each additional housemate^2^. Lifetime alcohol intake status (never/former/drinker) and smoking status (never/ex-smoker/smoker) were evaluated through trained researcher interviews and reviews of medical records^2^. The accuracy of the information was ensured by interviewing reliable informants^2^.

### Measurement of blood biomarkers

Blood samples were obtained by venipuncture in the morning after an overnight fast^2^. Albumin, glucose, high-density lipoprotein-cholesterol, low-density lipoprotein-cholesterol, iron, and uric acid were measured using a COBAS c702 analyzer and dedicated reagents (Roche Diagnostics, Manheim, Germany)^2^. Copper and zinc were measured using an ELAN DRC-e inductively coupled plasma-mass spectrometer (Perkin Elmer, Waltham, MA, USA)^2^.

### APOE genotyping

Apolipoprotein E (APOE) was genotyped using a Seeplex ApoE ACE genotyping kit (Seegene, Seoul, Korea)^2^. APOE ε4 allele (APOE4)-positivity was defined as the presence of at least one ε4 allele^2^.

### Statistical analysis

Between-group comparisons of continuous data (demographic and clinical data) employed one-way analysis of variance. Categorical data were analyzed using chi-square or Fisher exact tests. Multiple linear regression analyses were performed to examine the relationships between spicy level and cognition. The spicy level was the independent variable in each analysis; it was entered as a stratified categorical variable using the three categories. We tested three models and controlling for the covariates in a stepwise manner. The first model included age, sex, and education as covariates; the second model included those covariates plus APOE4-positivity, clinical diagnosis, VRS, body mass index (BMI), Geriatric Depression Scale score, physical activity, annual income status, alcohol intake, smoking, and other dietary styles (e.g., consumption of proteins, vegetables, fruits, salty foods, fatty foods, and fried foods); and the third model included the covariates in the second model plus albumin, glucose, high-density lipoprotein- and low-density lipoprotein-cholesterol, zinc, iron, copper, and uric acid. Prior to multiple linear regression analyses, data normalities were checked using the Kolmogorov–Smirnov test for dependent variables. For the sensitivity analyses, the same analyses were performed for participants with CN and MCI participants because the cognitive tests may be less discriminatory in CN individuals. To explore the moderating effects of age, sex, APOE4-positivity, VRS, BMI, and physical activity on the associations between spicy level and cognition, which was significant in the analyses described above, the same regression analyses were repeated with inclusion of two-way interaction terms between the spicy level and each of the six variables; these constituted additional independent variables. The level of statistical significance was set to a two-tailed *p* value < 0.05. All statistical analyses were performed using SPSS Statistics software ver. 28 (IBM Corp., Armonk, NY, USA).

### Ethics approval and consent to participate

This study protocol was approved by the institutional review board of the Hallym University Dongtan Sacred Heart Hospital and was conducted it in accordance with the recommendations of the current version of the Declaration of Helsinki. The subjects or their legal representatives gave informed consent.

## Results

### Characteristics of study sample

Table 1 summarizes the participants’ demographic and clinical characteristics. Of the 196 participants, 93, 58, and 45 belonged to the ‘not spicy’, ‘low spiciness’, and ‘high spiciness’ levels, respectively. There was a significant between-group difference in the level of spiciness by the memory score (*p* = 0.049). In contrast, there were no significant between group-differences in the level of spiciness by age, sex, education, APOE4-positivity, clinical diagnosis, vascular risks, depression, annual income, alcohol intake, smoking, physical activity, blood markers, dietary styles, or the other cognitive performances. No participants were in malnourished (serum levels of albumin < 3.5 g/dL^40^).

**Table 1:** Demographic and clinical characteristics of participants without dementia according to spicy level.

Characteristic	Overall	Categorized spicy level			*P*
		Not spicy	Low spiciness	High spiciness	
n	196	93	58	45	
Age, y	72.65 (5.95)	72.86 (6.35)	72.93 (5.92)	71.87 (5.15)	0.602^a^
Female, n (%)	138 (70.41)	63 (67.74)	39 (67.24)	36 (80.00)	0.275^b^
Education, y	9.62 (4.51)	9.45 (4.78)	10.26 (4.20)	9.16 (4.32)	0.414^a^
APOE4-positivity, n (%)	39 (19.90)	20 (21.51)	11 (18.97)	8 (17.78)	0.857^b^
MCI, n (%)	83 (42.35)	39 (41.94)	20 (34.48)	24 (53.33)	0.157^b^
VRS, %	23.98 (18.58)	24.19 (18.64)	25.29 (19.31)	21.85 (17.70)	0.643^a^
GDS score	10.92 (7.24)	11.25 (7.32)	9.79 (6.85)	11.69 (7.56)	0.352^a^
Annual income status					0.277^b^
< MCL, n (%)	25 (12.76)	16 (17.20)	5 (8.62)	4 (8.89)	
≥ MCL, < 2 × MCL, n (%)	62 (31.63)	24 (25.81)	20 (34.48)	18 (40.00)	
≥ 2 × MCL, n (%)	109 (55.61)	53 (56.99)	33 (56.90)	23 (51.11)	
Alcohol drink status, n (%)					0.211^b^
Never	107 (54.59)	53 (56.99)	34 (58.62)	20 (44.44)	
Former	34 (17.35)	13 (13.98)	13 (22.41)	8 (17.78)	
Drinker	55 (28.06)	27 (29.03)	11 (18.97)	17 (37.78)	
Smoking status, n (%)					0.619^c^
Never	149 (76.02)	69 (74.19)	42 (72.41)	38 (84.44)	
Former	39 (19.90)	19 (20.43)	14 (24.14)	6 (13.33)	
Smoker	8 (4.08)	5 (5.38)	2 (3.45)	1 (2.22)	
PASE total score	64.77 (46.21)	58.52 (39.95)	67.33 (47.90)	74.41 (54.46)	0.147^a^
BMI	24.83 (3.41)	24.66 (2.72)	24.70 (2.83)	25.35 (5.02)	0.507^a^
Albumin, g/dL	4.57 (0.26)	4.56 (0.27)	4.60 (0.27)	4.55 (0.23)	0.559^a^
Glucose, fasting, mg/dL	108.15 (19.94)	105.89 (18.41)	108.45 (18.57)	1122.33 (24.00)	0.207^a^
HDL-cholesterol, mg/dL	54.64 (12.96)	54.18 (12.84)	54.72 (12.08)	55.49 (14.46)	0.857^a^
LDL-cholesterol, mg/dL	94.51 (31.47)	90.92 (27.80)	93.07 (31.16)	104.38 (37.49)	0.064^a^
Copper, ug/dL	94.26 (16.73)	94.51 (17.09)	93.81 (17.00)	94.33 (15.96)	0.970^a^
Zinc, ug/dL	80.77 (11.15)	81.71 (10.88)	80.76 (12.86)	78.87 (9.15)	0.377^a^
Iron, ug/dL	99.18 (30.51)	102.98 (30.00)	91.71 (25.67)	101.20 (35.76)	0.079^a^
Uric acid, g/dL	4.70 (1.27)	4.70 (1.25)	4.75 (1.27)	4.65 (1.34)	0.918^a^
Other dietary styles, n (%)					
Protein					0.441^b^
Low (seldom)	71 (36.22)	39 (41.94)	19 (32.76)	13 (28.89)	
Moderate (sometimes)	81 (41.33)	33 (35.48)	25 (43.10)	23 (51.11)	
High (always)	44 (22.45)	21 (22.58)	14 (24.14)	9 (20.00)	
Vegetable					0.477^b^
Low (seldom)	26 (13.27)	11 (11.83)	11 (18.97)	4 (8.89)	
Moderate (sometimes)	45 (22.96)	20 (21.51)	15 (25.86)	10 (20.22)	
High (always)	125 (63.78)	62 (66.67)	32 (55.17)	31 (68.89)	
Fruits					0.202^b^
Low (seldom)	43 (21.94)	24 (25.81)	9 (15.52)	10 (22.22)	
Moderate (sometimes)	44 (22.45)	16 (17.20)	19 (32.76)	9 (20.00)	
High (always)	109 (55.61)	53 (56.99)	30 (51.72)	26 (57.78)	
Salty foods					0.670^b^
Low (seldom)	144 (73.47)	71 (76.34)	41 (70.69)	32 (71.11)	
Moderate (sometimes)	27 (13.78)	13 (13.98)	10 (17.24)	4 (8.89)	
High (always)	23 (11.73)	9 (9.68)	7 (12.07)	7 (15.56)	
Fatty foods					0.967^c^
Low (seldom)	154 (78.57)	74 (79.57)	45 (77.59)	35 (77.78)	
Moderate (sometimes)	27 (13.78)	13 (13.98)	8 (13.79)	6 (13.33)	
High (always)	13 (6.63)	6 (6.45)	5 (8.62)	2 (4.44)	
Fried foods					0.346^b^
Low (seldom)	148 (75.51)	70 (75.27)	42 (72.41)	36 (80.00)	
Moderate (sometimes)	24 (12.24)	15 (16.13)	7 (12.07)	2 (4.44)	
High (always)	21 (10.71)	8 (8.60)	8 (13.79)	5 (11.11)	
Cognitive performance					
Total score	69.98 (15.61)	70.32 (13.95)	72.16 (15.80)	66.47 (18.20)	0.179^b^
Memory score	35.10 (9.48)	35.68 (9.39)	36.47 (8.79)	32.13 (10.10)	0.049^b^
Non-memory score	34.25 (6.62)	34.22 (5.79)	34.74 (7.08)	33.69 (7.64)	0.726^b^

Data are expressed as mean (standard deviation), unless otherwise indicated.

*MMSE* mini-mental state examination, *APOE4* apolipoprotein E ε4 allele, *MCI* mild cognitive impairment, *VRS* vascular risk score, *GDS* geriatric depression scale, *MCL* minimum cost of living, *BMI* body mass index, *PASE* physical activity scale for the elderly.

^a^By one-way analysis of variance.

^b^By chi-square test.

^c^By Fisher exact test.

### Associations between strength of spicy food and cognition

**Figure 1:**
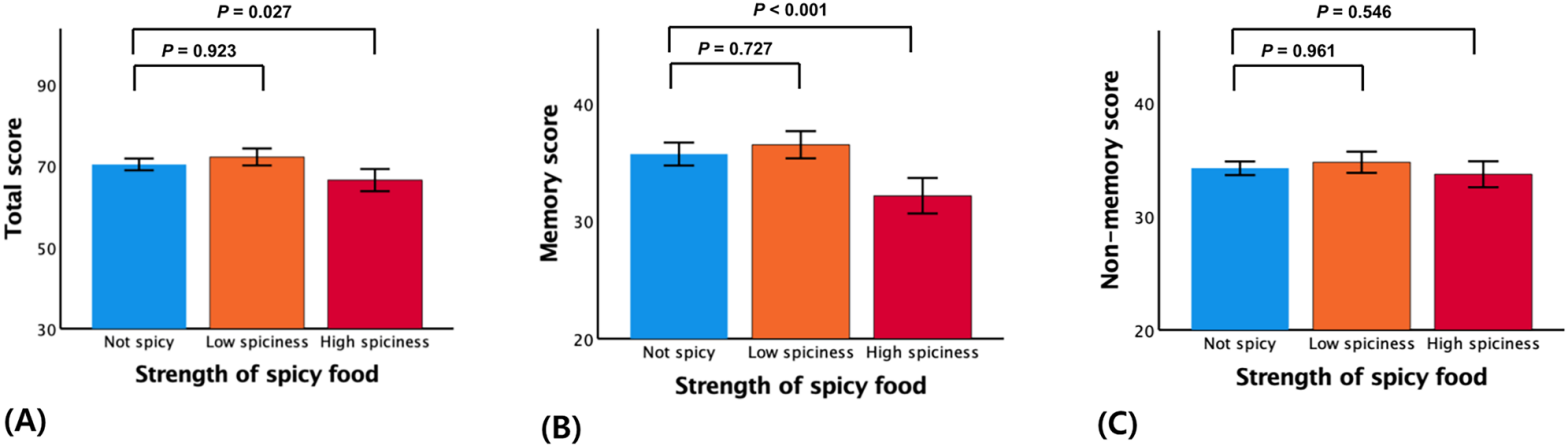
Bar plots of the association between stratified spicy levels and (**A**) total score, (**B**) memory score, or (**C**) non-memory score in non-demented older participants. Footnotes: Error bars indicate standard errors. Multiple linear regression analyses were performed after adjusting for all confounders.

TS as global cognitive score was significantly differed among the stratified spicy strength levels. A high spiciness level was significantly associated with lower TS compared to the not-spicy level (*β* − 0.122, *p* = 0.027), but low spiciness level was not (Table 2 and Fig. 1A). Regarding to cognitive domains, *i.e.,* memory and non-memory domains, memory score was significantly differed among the stratified spicy strength levels, but non-memory score did not. A high spiciness level was also significantly associated with lower memory score compared to the not-spicy level (*β* − 0.167, *p* < 0.001), but low spiciness level was not (Table 2 and Fig. 1B,C).

**Table 2:** Results of multiple linear regression analyses concerning associations of spicy levels with cognition in non-demented older adults.

	Total score		Memory score		Non-memory score	
	$\beta$	*p*	$\beta$	*p*	$\beta$	*p*
Model 1						
High spiciness	− 0.136	**0.039**	− 0.201	**0.003**	− 0.041	0.553
Low spiciness	0.019	0.768	0.006	0.928	0.004	0.954
Not spicy	Reference		Reference		Reference	
Model 2						
High spiciness	− 0.130	**0.016**	− 0.169	**< 0.001**	− 0.053	0.403
Low spiciness	− 0.017	0.740	− 0.025	0.590	− 0.013	0.839
Not spicy	Reference		Reference		Reference	
Model 3						
High spiciness	− 0.122	**0.027**	− 0.167	**< 0.001**	− 0.040	0.546
Low spiciness	− 0.005	0.923	− 0.016	0.727	0.003	0.961
Not spicy	Reference		Reference		Reference	

The first model included age, sex, and education as covariates; the second model included those covariates plus APOE4-positivity, clinical diagnosis, VRS, BMI, GDS, PASE, annual income status, alcohol intake, smoking, and other dietary styles (protein, vegetable, fruit, salty- and fatty- and fried-food); and, the third model included the covariates in the second model plus albumin, glucose, HDL- and LDL-cholesterol, zinc, iron, copper, and uric acid.

*APOE4* apolipoprotein E ε4 allele, *VRS* vascular risk score, *BMI* body mass index, *GDS* geriatric depression scale, *PASE* physical activity scale for the elderly.

### Moderation of the associations between spicy level and cognition

**Figure 2:**
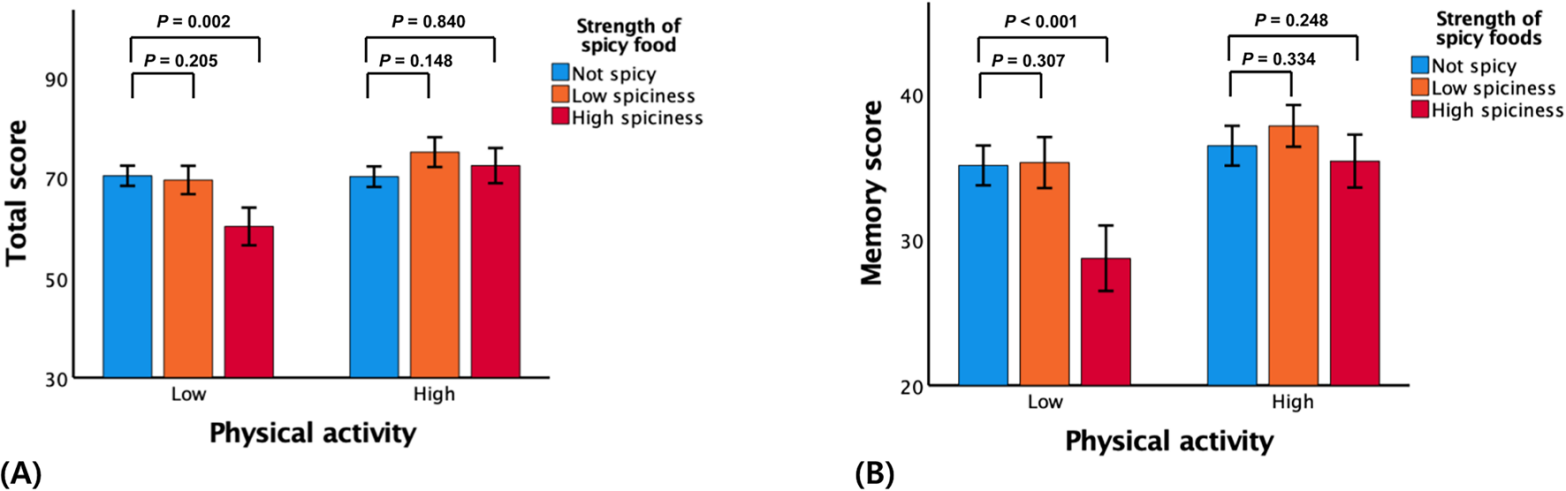
Bar plots of the association between stratified spicy levels and (**A**) total score or (**B**) memory score in non-demented older participants according to subgroup (low and high physical activity levels). Footnotes: Error bars indicate standard errors. Multiple linear regression analyses were performed after adjusting for all confounders.

The spicy level × physical activity interaction was significant in terms of the TS (*β* 0.336, *p* = 0.001) and memory score (*β* 0.209, *p* = 0.029), indicating that physical activity moderated the association between the spicy level and global and memory cognition (Table 3). Further subgroup analyses showed that a high spiciness level was significantly associated with a low TS (*β* − 0.222, *p* = 0.002) and memory score (*β* − 0.254, *p* < 0.001) in the low physical activity subgroup but not in the high physical activity subgroup (Table 4 and Fig. 2A,B). The interactions of spicy level with age, sex, APOE4-positivity, VRS, and BMI were not statistically significant (Table 3).

**Table 3:** Results of multiple linear regression analyses, including the interaction terms between the spicy level strata and age (or sex, or APOE4-positivity, or VRS, or BMI, or physical activity) in terms of predicting total score or memory score.

	Total score		Memory score	
	$\beta$	*P*	$\beta$	*P*
High spiciness	0.627	0.534	0.453	0.536
Low spiciness	1.488	0.058	1.107	0.058
Age	− 0.188	0.022	− 0.101	0.093
High spiciness × age	− 0.753	0.454	− 0.616	0.399
Low spiciness × age	− 1.477	0.060	− 1.138	0.052
High spiciness	− 0.103	0.094	− 0.153	0.006
Low spiciness	− 0.034	0.591	− 0.040	0.480
Sex	− 0.122	0.188	− 0.050	0.545
High spiciness × sex	− 0.062	0.318	− 0.044	0.429
Low spiciness × sex	0.058	0.419	0.048	0.462
High spiciness	− 0.101	0.101	− 0.174	0.002
Low spiciness	0.016	0.795	0.001	0.987
APOE4-positivity	0.007	0.925	− 0.062	0.351
High spiciness × APOE4-positivity	− 0.053	0.424	0.018	0.756
Low spiciness × APOE4-positivity	− 0.048	0.494	− 0.046	0.470
High spiciness	− 0.140	0.124	− 0.287	< 0.001
Low spiciness	− 0.084	0.351	− 0.079	0.325
VRS	− 0.040	0.592	− 0.115	0.089
High spiciness × VRS	0.022	0.812	0.156	0.065
Low spiciness × VRS	0.105	0.279	0.084	0.332
High spiciness	− 0.129	0.734	− 0.280	0.411
Low spiciness	0.250	0.610	− 0.041	0.927
BMI	0.091	0.355	− 0.003	0.974
High spiciness × BMI	0.005	0.990	0.118	0.737
Low spiciness × BMI	− 0.258	0.601	0.025	0.956
High spiciness	− 0.348	< 0.001	− 0.307	< 0.001
Low spiciness	− 0.104	0.248	− 0.073	0.375
PASE	− 0.091	0.263	− 0.026	0.727
High spiciness × PASE	0.336	**0.001**	0.209	**0.029**
Low spiciness × PASE	0.156	0.128	0.090	0.336

Multiple linear regression model included spicy food level strata, age (or sex, or APOE4-positivity, or VRS, or BMI, or PASE) and the interaction between spicy food level strata and age (or sex, or APOE4-positivity, or VRS, or BMI, or PASE) treated as the independent variables; for all potential confound factors were treated as covariates; and total score or memory score treated as the dependent variable.

*APOE4* apolipoprotein ε4 allele, *VRS* vascular risk score, *BMI* body mass index, *PASE* physical activity scale for the elderly, *PASE* physical activity scale for the elderly.

**Table 4:** Results of the multiple linear regression analyses concerning associations of spicy level strata with memory score according to physical activity subgroup.

	High physical activity				Low physical activity			
	Total score		Memory score		Total score		Memory score	
	$\beta$	*p*	$\beta$	*p*	$\beta$	*p*	$\beta$	*p*
Model 1								
High spiciness	− 0.007	0.947	− 0.126	0.230	− 0.232	**0.009**	− 0.250	**0.005**
Low spiciness	0.104	0.306	0.029	0.780	− 0.030	0.727	0.015	0.863
Not spicy	Reference		Reference		Reference		Reference	
Model 2								
High spiciness	0.043	0.638	− 0.086	0.279	− 0.273	**< 0.001**	− 0.266	**< 0.001**
Low spiciness	0.137	0.128	0.085	0.264	− 0.130	0.058	− 0.083	0.191
Not spicy	Reference		Reference		Reference		Reference	
Model 3								
High spiciness	0.019	0.840	− 0.093	0.248	− 0.222	**0.002**	− 0.254	**< 0.001**
Low spiciness	0.134	0.148	0.075	0.334	− 0.088	0.205	− 0.069	0.307
Not spicy	Reference		Reference		Reference		Reference	

The first model included age, sex, and education as covariates; the second model included those covariates plus APOE4-positivity, clinical diagnosis, VRS, BMI, GDS, PASE, annual income status, alcohol intake, smoking, and other dietary styles (protein, vegetable, fruit, salty- and fatty- and fried-food); and, the third model included the covariates in the second model plus albumin, glucose, HDL- and LDL-cholesterol, zinc, iron, copper, and uric acid.

*APOE4* apolipoprotein E ε4 allele, *VRS* vascular risk score, *BMI* body mass index, *GDS* geriatric depression scale, *PASE* physical activity scale for the elderly.

### Sensitivity analyses

The sensitivity analysis of the participants with MCI and CN showed similar results for memory score, but not for TS (Tables 5 and 6). A high spiciness level was significantly associated with a lower memory score compared to not-spicy level among participants with MCI (*β* − 0.325, *p* = 0.002) and CN (*β* − 0.238, *p* = 0.013), but a low spiciness level was not.

**Table 5:** Results of multiple linear regression analyses concerning associations of spicy levels with cognition in older adults with mild cognitive impairment.

	Total score		Memory score		Non-memory score	
	$\beta$	*p*	$\beta$	*p*	$\beta$	*p*
Model 1^a^						
High spiciness	− 0.190	0.078	− 0.196	0.058	− 0.115	0.313
Low spiciness	− 0.116	0.276	− 0.082	0.417	− 0.081	0.471
Not spicy	Reference		Reference		Reference	
Model 2^b^						
High spiciness	− 0.292	**0.008**	− 0.300	**0.004**	− 0.192	0.099
Low spiciness	− 0.113	0.279	− 0.060	0.544	− 0.054	0.629
Not spicy	Reference		Reference		Reference	
Model 3^c^						
High spiciness	− 0.303	**0.003**	− 0.325	**0.002**	− 0.168	0.111
Low spiciness	− 0.165	0.074	− 0.089	0.336	− 0.111	0.256
Not spicy	Reference		Reference		Reference	

The first model included age, sex, and education as covariates; the second model included those covariates plus APOE4-positivity, VRS, BMI, GDS, PASE, annual income status, alcohol intake, smoking, and other dietary styles (protein, vegetable, fruit, salty- and fatty- and fried-food); and, the third model included the covariates in the second model plus albumin, glucose, HDL- and LDL-cholesterol, zinc, iron, copper, and uric acid.

*APOE4* apolipoprotein E ε4 allele, *VRS* vascular risk score, *BMI* body mass index, *GDS* geriatric depression scale, *PASE* physical activity scale for the elderly.

**Table 6:** Results of multiple linear regression analyses concerning associations of spicy levels with cognition in cognitive normal older adults.

	Total score		Memory score		Non-memory score	
	$\beta$	*p*	$\beta$	*p*	$\beta$	*p*
Model 1						
High spiciness	0.012	0.880	− 0.138	0.090	0.098	0.267
Low spiciness	0.074	0.351	− 0.015	0.848	0.050	0.569
Not spicy	Reference		Reference		Reference	
Model 2						
High spiciness	− 0.026	0.746	− 0.210	**0.013**	0.084	0.353
Low spiciness	0.064	0.418	0.001	0.992	0.063	0.478
Not spicy	Reference		Reference		Reference	
Model 3						
High spiciness	− 0.050	0.576	− 0.238	**0.013**	0.068	0.491
Low spiciness	0.048	0.564	− 0.021	0.809	0.044	0.628
Not spicy	Reference		Reference		Reference	

The first model included age, sex, and education as covariates; the second model included those covariates plus APOE4-positivity, VRS, BMI, GDS, PASE, annual income status, alcohol intake, smoking, and other dietary styles (protein, vegetable, fruit, salty- and fatty- and fried-food); and, the third model included the covariates in the second model plus albumin, glucose, HDL- and LDL-cholesterol, zinc, iron, copper, and uric acid.

*APOE4* apolipoprotein E ε4 allele, *VRS* vascular risk score, *BMI* body mass index, *GDS* geriatric depression scale, *PASE* physical activity scale for the elderly.

## Discussion

This study of adults without dementia showed that the consumption of food with a high level of spiciness was associated with greater AD-related cognition as indicated by the memory score, i.e., episodic memory; this association was moderated by the effect of physical activity.

Previous studies have suggested that the consumption of spicy food is inversely associated with AD or related cognitive dysfunction^6–8^. Preclinical studies using AD-transgenic mice^6,8^ indicated that the consumption of capsaicin, the pungent ingredient in chili peppers, reduces brain β-amyloid (Aβ) burden and rescues cognitive decline in AD-transgenic mice^6^. Daily capsaicin treatment attenuated cognitive and synaptic function, whereas a specific agonist of the transient receptor potential vanilloid 1 antagonist treatment did not, indicating that the activation of transient receptor potential vanilloid 1 by capsaicin rescues cognitive deficit in AD transgenic mice^8^. A human study also indicated that the consumption of a capsaicin-rich diet was associated with better cognition and lower serum Aβ levels in participants aged ≥ 40 years^7^.

In contrast, we found that a high spiciness level increased AD or related cognitive decline in older adults without dementia. This finding is consistent with the results in a longitudinal cohort study of 4582 adults over 15 years^9^, which revealed that higher chili intake (> 50 g/day) was positively associated with lower cognitive scores (as measured by the global cognitive score and self-reported memory loss) compared with non-consumers of chili. The authors of the longitudinal cohort study emphasized the differences between their findings and the finding reported in the human study noted above^7^; they presumed that the differences were related to an age discrepancy in terms of the quantity and frequency of chili consumption (i.e., participants with a high chili intake were younger than participants who did not consume chili)^9^. In the present study, all participants were older adults (mean age [SD] = 72.7 [6.0] years); there was no age difference between the spiciness groups (*p* = 0.602) (Table 1). Thus, unlike in a former study^7^, age-related differences in spiciness did not seem to affect our findings. A preclinical study demonstrated the capsaicin-induced neurotoxic effect of high capsaicin intake^12^. The authors of that study suggested that capsaicin exerts an excitatory action by activating calcium ion channels, which causes a prolonged increase in intracellular calcium ion concentrations^10^ that is associated with a glutamate excitotoxic effect in neurons^10^; these interactions are the first events in a sequence that ultimately leads to cell death^12,13^. Furthermore, high doses of capsaicin have been used as an analgesic agent for pain management through chemical denervation of sensory nerves^11^.

The physical activity status of our participants moderated the association between the spicy level and AD-related cognitive decline, as indicated by the memory score, i.e., episodic memory. A significant association was detected between a high spiciness level and worse episodic memory performance in participants with low physical activity, but not in participants with high physical activity. This finding may reflect the interactions among spicy levels, physical activity, and AD-related cognitive decline through neurotoxic effects. A preclinical study showed that physical activity increased mitochondrial glutamate metabolism in the mouse cerebral cortex, thereby lowing excessive brain glutamate and reducing its toxicity^15^. Additionally, many studies have shown that physical activity has a neuroprotective effect on the brain through various mechanisms^14^ and may protect the brain from neurotoxic stimuli^41^. Therefore, the toxicity caused by a high spicy level was offset by these benefits of high physical activity but not by low physical activity.

### Strengths and limitations

To our knowledge, this is the first study to show an association between spicy level and AD-related cognition in humans using international and Korean official standard heat units (i.e., SHU, ppm, and GHU) for measuring the spicy levels, as well as the moderating effect of the spicy level on the association. The findings did not change after controlling for all potential confounders. The results were confirmed by sensitivity analysis after excluding participants who were CN and MCI. Our study had some limitations. First, because this was a cross-sectional study, causal relationships could not be inferred. Long-term prospective studies are needed. Second, there were raising some concerns about recall bias as we obtained the information on preference for spicy foods through clinical interviews. Approximately 42.3% of the study participants were diagnosed with MCI, which could raise some concerns about the accuracy of self-report on the preference for spicy food. However, participants with MCI have problems for their recent memory their remote memory is very well-preserved^42^. Thus, it is not likely that participants with MCI reported their preference for spicy food more erroneously because the self-report for their preference for spicy food mainly depends on remote memory rather than recent memory. As a lifestyle, dietary habits are not easily changed and persist in their lifetime, so they could report it accurately. In addition, even when we controlled for the clinical diagnosis as an additional covariate in Model 2, the results were still very similar. Moreover, the accuracy of the information of preference for spicy food was verified by reliable informants. Finally, we lacked adequate data on the frequency, amount and duration of cumulative exposure to spicy food; we cannot draw any significant associations. Further studies are needed.

## Conclusion

Our findings suggest that spicy food intake is predictive of AD-related cognitive decline, i.e., episodic memory; this relationship is worsened by physically inactive lifestyle. Clinicians may need to monitor the consumption of spicy foods in older adults, along with their physical activity, to prevent AD or cognitive decline.

## Data Availability

The study data are not freely accessible because the IRB of the Hallym University Dongtan Sacred Heart Hospital prevents public sharing of such data for privacy reasons. However, the data are available on reasonable request after IRB approval. Requests for data access can be submitted to an independent administrative coordinator by e-mail (yoon4645@gmail.com).
